# Testosterone-induced adult neurosphere growth is mediated by sexually-dimorphic aromatase expression

**DOI:** 10.3389/fncel.2015.00253

**Published:** 2015-07-06

**Authors:** Mark I. Ransome, Wah Chin Boon

**Affiliations:** ^1^Steroid Neurobiology Laboratory, The Florey Institute of Neuroscience and Mental HealthParkville, VIC, Australia; ^2^Department of Anatomy and Developmental Biology, Monash UniversityClayton, VIC, Australia

**Keywords:** neurosphere, sex hormones, aromatase, oxidative stress, neural stem cell, proliferation, estradiol, testosterone

## Abstract

We derived adult neural stem/progenitor cells (NSPCs) from the sub-ventricular zone of male and female mice to examine direct responses to principal sex hormones. In the presence of epidermal growth factor (EGF) and fibroblast growth factor-2 (FGF2) NSPCs of both sexes expressed nestin and sox2, and could be maintained as neurospheres without addition of any sex hormones. The reverse was not observed; neither testosterone (T), 17β-estradiol (E_2_) nor progesterone (P_4_) was able to support neurosphere growth in the absence of EGF and FGF2. Ten nanomolar T, E_2_ or P_4_ induced nestin(+) cell proliferation within 20 min and enhanced neurosphere growth over 7 days irrespective of sex, which was abolished by Erk inhibition with 20 μM U0126. Maintaining neurospheres with each sex hormone did not affect subsequent neuronal differentiation. However, 10 nM T, E_2_ or P_4_ added during differentiation increased βIII tubulin(+) neuron production with E_2_ being more potent compared to T and P_4_ in both sexes. Androgen receptor (AR) inhibition with 20 μM flutamide but not aromatase inhibition with 10 μM letrozole reduced basal and T-induced neurosphere growth in females, while only concurrent inhibition of AR and aromatase produced the same effect in males. This sex-specific effect was supported by higher aromatase expression in male neurospheres compared to females measured by Western blot and green fluorescent protein (GFP) reporter. Ten micromolar menadione induced oxidative stress, impaired neurosphere growth and up-regulated aromatase expression in both sexes. However, under oxidative stress letrozole significantly exacerbated impaired neurosphere growth in males only. While both E_2_ and T could prevent oxidative stress-induced growth reduction in both sexes, the effects of T were dependent on innate aromatase activity. We show for the first time that intrinsic androgen and estrogen signaling may impact the capacity of NSPCs to produce neural progenitors under pathological conditions of oxidative stress.

## Introduction

The central edict that adult mammalian brains do not produce new neurons persevered up until relatively recently. The eventual acceptance that neural stem/progenitor cells (NSPCs) do exist in adult mammalian brains was a watershed in our view of the brain’s regenerative capacity (Gross, 2000; Ransome, 2012b; Faigle and Song, 2013). Adult NSPCs are largely thought to be anatomically restricted; the sub ventricular zone (SVZ) of the lateral ventricles is one major site where NSPCs continue to produce new neurons (Gould, 2007; Ming and Song, 2011). Under otherwise normal physiology, SVZ NSPCs give rise to new neurons in the process of neurogenesis that populate the olfactory bulb (Ming and Song, 2011). However, pathological conditions such as traumatic brain injury and stroke, appear capable of driving SVZ NSPCs into populating injured cortex with new neurons (Sawada and Sawamoto, 2013). Variably this process has been coined injury-induced neurogenesis and generated significant interest in understanding factors that regulate adult NSPC properties.

The innate efficacy of injury-induced neurogenesis is limited by the apparent failure of NSPCs to produce persistent neurons in the injured region. At present this failure means adult NPSCs represent a potential endogenous repair mechanism yet to be harnessed. The objective of therapeutically exploiting this potential underscores intense efforts to understand the mechanisms that govern NSPC regulation. A significant step toward understanding cell-intrinsic regulation was achieved when NSPCs were isolated from adult SVZ and propagated *in vitro* (Reynolds and Weiss, 1992). Cultured adult NSPCs derived from the SVZ can be propagated as floating cell aggregates termed neurospheres, within which the key self-renewal and multipotent NSPC properties are maintained. The neurosphere assay has proven to be a powerful tool in examining molecular control of these key NSPC properties with the aim of promoting the endogenous capacity of the injured brain to self-repair (Bellenchi et al., 2013).

Successful therapeutic targeting of adult NSPCs requires fundamental gaps in our current knowledge to be addressed (Ming and Song, 2011). One key focus is on the potential sexually-dimorphic regulation of adult NSPCs. The primary sex hormones 17β-estradiol (E_2_) and testosterone (T) were amongst the first physiological factors observed to influence adult NSPCs (Tanapat et al., 1999; Galea, 2008; Ransome, 2012b). Disparity in gonad-derived sex hormone levels is a principal determinant of sexual differentiation (Arnold, 2009) and therefore circulating E_2_ and T could be prime mediators of sexually-dimorphic NSPC regulation. However, a stable framework describing sex-specific adult NSPC responses to sex hormones remains elusive (Lagace et al., 2007; Tatar et al., 2013). Genes encoding key enzymes involved in sex hormone synthesis are expressed in the adult brain (Mensah-Nyagan et al., 1999), which provides the capacity to metabolize circulating sex hormones that freely enter the central nervous system. This capacity confounds the ability to attribute changing circulating sex hormone levels with functional changes in NSPCs. A prime example is the localized brain expression of the *Cyp19* gene encoding the aromatase enzyme responsible for E_2_ synthesis (Boon et al., 2010; Stanić et al., 2014). Aromatase synthesizes E_2_ using T as a direct precursor; hence cells expressing aromatase can convert circulating T directly into E_2_, thereby activating androgen receptors (AR) or estrogen receptors (ER), respectively. Limited reports using adult neurospheres to examine direct cell effects suggest NSPCs express AR and ER and therefore in principal can be influenced directly by sex hormones (Brännvall et al., 2005; Ray et al., 2008; Waldron et al., 2010a,b). In this study we isolated NSPCs from both female and male adult mice to examine if cell-direct effects of sex hormones at equal molar concentrations were determined by genetic sex.

## Materials and Methods

### Animals

All experimental procedures performed in this study conformed to the Australian National Health and Medical Research Council published code of practice, and were approved by the Animal Ethics Committee of The Florey Institute of Neuroscience and Mental Health (#12-086). C57Bl6 male and female mice 8–10 weeks of age were purchased from the Animal Resources Centre (Canning Vale, WA, and Australia) and allowed to acclimatize for 1 week in the bio-resource animal facility at the Florey Institute for Neuroscience and Mental Health (FINMH) before being used to derive adult neural stem progenitor cells. Male and female mice from the transgenic CYP19A1-EGFP BAC-mouse strain (FVB/N) were obtained at 8–10 weeks of age from stocks maintained within the FINMH bio-resource animal facility and described previously (Stanić et al., 2014).

### Neurosphere Culture

NSPCs were isolated from the SVZ of 8–10 week old mice as described (Scott et al., 2006) and cultured in neurosphere proliferation media consisting of Advanced Dulbecco’s Modified Eagle’s Medium (DMEM):F12 (Invitrogen, Life Technologies, Mulgrave, VIC, Australia) supplemented with 100 U/ml penicillin/streptomycin, 0.25 μg/ml Funlzone® (Antibiotic-Antimycotic, Gibco), 20 ng/ml epidermal growth factor (EGF; Peprotec, NJ, USA), 10 ng/ml fibroblast growth factor 2 (FGF2; Peprotec), 0.1 mg/ml apo-transferrin (Sigma-Aldrich, Castle Hill, NSW, Australia), 5 μg/ml insulin, 100 μM putrescene (Sigma-Aldrich), 30 nM sodium selenite (Sigma-Aldrich), 2 mM GlutaMax™ (Invitrogen). Primary neurospheres were then maintained by weekly passaging through dissociating in TrypLE™ (Gibco) with 200 U/ml DNase I (Roche Products Pty Ltd, NSW, Australia). Viable cells were counted using a hemocytometer and trypan blue dye exclusion and seeded at 50,000 cells/ml in proliferation media in either T75 cm^2^ or T25 cm^2^ Nunc flasks (Thermo Scientific, MA, USA) and kept humidified at 37°C with 5% CO_2_. Each line was maintained for experimentation until passage 10.

### Neurosphere Growth Assay/Proliferation Assay

NSPCs were seeded at 20,000 cells/ml of proliferation media in 12 well culture plates (1.5 ml per well) and maintained ± treatments for times indicated. Neurospheres were then dissociated in TrypLE/DNase I and viable cells counted using a hemocytometer and trypan blue dye exclusion and the percentage increase in cells determined. Results derived from three independent male and three independent female cell lines are expressed as mean ± SEM. For acute experiments NSPCs were seeded at 50,000 cells/ml of proliferation media in T25 cm^2^ flasks and grown for 4 days then collected by centrifugation and resuspended in 8 ml of proliferation media. Aliquots (500 μl) were then added to 8-well chamber slides (Millipore, Bayswater, VIC, Australia) coated with 50 ng/ml poly-D-lysine (Sigma-Aldrich) and 20 ng/ml mouse laminin (Invitrogen) for 30 min before addition of treatments for 20 min. Cells were then washed in ice-cold 0.1 M phosphate buffered saline (PBS) then fixed in 4% paraformaldehyde (PFA) on ice for 20 min. Immunocytochemistry was then performed; cells were blocked in 0.1 M PBS containing 5% normal donkey serum (Millipore) and 0.1% triton-X100 (Sigma-Aldrich) for 60 min at 20°C then incubated with primary antibodies, rabbit anti-Ki67 (1:300, DKSH Australia Pty Ltd., VIC, Australia), mouse anti-nestin (1:300, R and D Systems, MN, USA) for 3 h, washed in PBS then incubated in secondary antibodies, Alexa fluor 488-conjugated donkey anti-rabbit (1:400, Molecular Probes, Life Technologies) and Alexa fluor 555-conjugated donkey anti-mouse (1:400, Molecular Probes) then counterstained with 4′,6-Diamidino-2-Phenylindole (1:2000, DAPI, Sigma-Aldrich) for 10 min and coverslipped in Fluoromount (DAKO, North Sydney, NSW, Australia). Digital images were taken using an Olympus BX61 microscope with an UPlan FL N 20 × objective lens (Olympus, Notting Hill, VIC, Australia) in 15 randomly chosen areas per chamber well per condition and coded. Coded images were then imported into Image J software (NIH, Bethesda, MD, USA) and randomly chosen neurospheres were analyzed if they were a clearly demarcated mono layer, containing 25–40 DAPI/nestin(+). Results are expressed as percentage nestin/DAPI(+) cells co-labeled with Ki67, mean ± SEM from three independent female and three independent male cell lines. Immunocytochemical assessment of adult NSPC markers was also performed as described above using mouse anti-nestin and goat anti-SOX2 (both 1:300, R and D Systems) followed by Alexa fluor 555-conjugated donkey anti-goat, Alexa fluor 488-conjugated donkey anti-mouse and DAPI. Between 40–50 neurospheres per condition were assessed for nestin/sox2/DAPI co-labeling.

### Neurosphere Growth/Proliferation Assay Treatments

Pilot proliferation assays showed no change in neurosphere growth when 0.01% v/v ethanol or dimethyl sulfoxide (DMSO, Sigma-Aldrich) were added to the media compared to proliferation media alone. T, E2 and P_4_ were obtained from Sigma-Aldrich and 1 mM stock solutions made in 100% ethanol. For each assay 1 mM stock solutions were serially diluted in media to a final concentration of 10 nM (except where indicated in results) with a final ethanol concentration of 0.001%. Flutamide and letrozole (Sigma-Aldrich) were made to 10 mM stock solutions in 100% ethanol and DMSO, respectively and serially diluted in media to a final concentration of 10 and 20 μM, respectively. Ten millimolar Menadione (Sigma-Aldrich) stock solutions in 100% ethanol were made regularly and protected from light before serial dilution in media to a final concentration of 10 μM. The Erk inhibitor U0126 (Cell Signaling Technologies Inc., MA, USA) was used at a final concentration of 20 μM.

### Neurosphere Neuronal Differentiation Assay

Dissociated neurospheres were seeded at 50,000 cells/ml in 6 ml proliferation media ± treatments in T25 cm^2^ flasks and grown for 4 days. Neurospheres were then collected by centrifugation and resuspended in 4 ml of fresh proliferation media then aliquoted into poly-D-lysine/laminin coated 8-well chamber slides (Millipore) 500 μl/chamber. After 6 h proliferation media was replaced with differentiation media consisting of DMEM:F12 supplemented with 2 mM Glutamax, 1% Antibiotic-Antimycotic (all Gibco), 10 μM retinoic acid (Sigma-Aldrich), 0.1 mg/ml apo-transferrin (Sigma-Aldrich) and 5 μg/ml insulin, ± treatments as indicated. After 2 days cultures were washed with ice-cold PBS then fixed on ice with 4% PFA for 20 min. Immunocytochemistry was then performed; cells were blocked in 0.1 M PBS containing 5% normal donkey serum (Millipore) and 0.1% triton-X100 (Sigma-Aldrich) for 60 min at 20°C then incubated with mouse anti-βIII tubulin (1:1000, Promega Corp., WI, USA) for 3 h, washed in PBS then incubated in Alexa fluor 555-conjugated donkey anti-mouse (1:400, Molecular Probes), then counterstained with DAPI (1:2000, Sigma-Aldrich) for 10 min and coverslipped in Fluoromount (DAKO). Digital images were taken using an Olympus BX61 microscope with an UPlan FL N 20 × objective lens (Olympus) in 15 randomly chosen areas per chamber well per condition and coded. Image J software (Bethesda, MD, USA) was used to count and the number of βIII-tubulin(+) cells as a proportion of total DAPI(+) cells per image. Results are expressed as mean ± SEM from three independent female and three independent male cell lines.

### Western Blot Analyses

Control neurospheres and those treated with 10 μM menadione for 3 h were collected by centrifugation and lysed on ice for 30 min in standard lysis buffer with added protease inhibitor cocktail (Sigma) before being cleared by centrifugation to generate whole-cell lysates. Protein concentration was determined by the Card Direct™ method (Millipore) and 30 μg of protein per sample were resolved under reducing conditions on 8–16% polyacrylaminde gels for 1 h at 90V then transferred to an Immobilon PVDF-FL membrane (Millipore) for 3 h at 200 mA. After being washed in 0.1 M tris-buffered saline (TBS) membranes were blocked with 5% skim milk powder in TBS for 1 h at 20°C then incubated in rabbit anti-aromatase (1:500, Sigma Aldrich) or rabbit anti-AR (N20, 1:500, Santa Cruz Biotechnology, Inc., TX, USA) diluted in TBS overnight at 4°C. Membranes were washed again then incubated in donkey anti-rabbit IRDye680 (1:5000, Li-Cor, Lincoln, NE, USA) for 3 h at 20°C. Digital images of immunoreactive bands were captured using an Odyssey Classic near infrared detection system. Membranes were then re-probed with mouse anti-βactin (1:5000, Sigma) then donkey anti-mouse IRDye800 (1:5000, Li-Cor) and digital images captured. Gray-scale digital images were subject to densitometry using Image J software (NIH, Bethesda, MD, USA). Optical densities of aromatase and AR immunoreactive bands were measured and normalized to respective βactin bands. Results are expressed as mean ± SEM from three independent female and three independent male cell lines.

### Statistical Analysis

All statistical analyses were performed using GraphPad Prism 5.0 software (GraphPad Software, Inc., San Diego, CA, USA). Student’s un-paired 2-tailed *t*-test and multiple group comparisons using one and two way ANOVA were used with a Tukey’s multiple comparison test for ANOVA. In all cases, significance was set at *P* < 0.05.

## Results

### Direct Androgen Signaling in NSPCs

**Figure 1:**
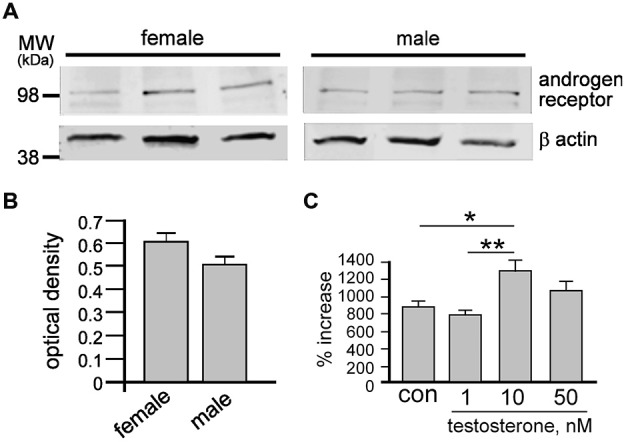
**After 7 days under control conditions, total cell protein was obtained from female and male neurospheres then resolved by sodium dodecyl sulfate polyacrylamide gel electrophoresis (SDS-PAGE) and probed for androgen receptor (AR) and β actin (A).** Quantitative densitometry analyses demonstrate similar protein levels of androgen receptor in both female and male neurospheres **(B)**. Female neurospheres were grown over 7 days ± 1 nM, 10 nM or 50 nM testosterone (T) then dissociated into single cells and counted **(C)**. While 1 nM T had no effect, both 10 and 50 nM T significantly increased neurosphere growth. Results show mean ± SEM, *p < 0.05, **p < 0.01, one-way ANOVA with Tukey’s multiple comparison test.

To clarify whether T could directly affect NSPCs, we analyzed AR protein levels in female and male neurospheres by quantitative Western blot (Figure 1A). AR protein was found in neurospheres of both sexes with densitometry analyses and statistical *t*-test showing no significant differences in levels between sexes (Figure 1B). Based on previous work showing effects of T derivatives on adult female neurospheres (Brännvall et al., 2005), we next examined different doses of exogenous T (1 nM, 10 nM or 50 nM) on female neurospheres over 7 days and compared changes in growth (Figure 1C). One-way ANOVA analyses showed a significant main effect of treatment, *F*_(3,8)_ = 7.81, *p* < 0.01, with Tukey’s multiple comparisons test showing significant increases induced by 10 nM T compared to control, *p < 0.05, and 10 nM T compared to 1 nM T, **p < 0.01. Based on these data we proceeded to compare the equal molar effects of T, P_4_ and E_2_ on male and female NSPCs using a final concentration of 10 nM.

### Sex Hormone Effects on Neural Stem Cell Markers, Neurogenic Potential and Neurosphere Maintenance

**Figure 2:**
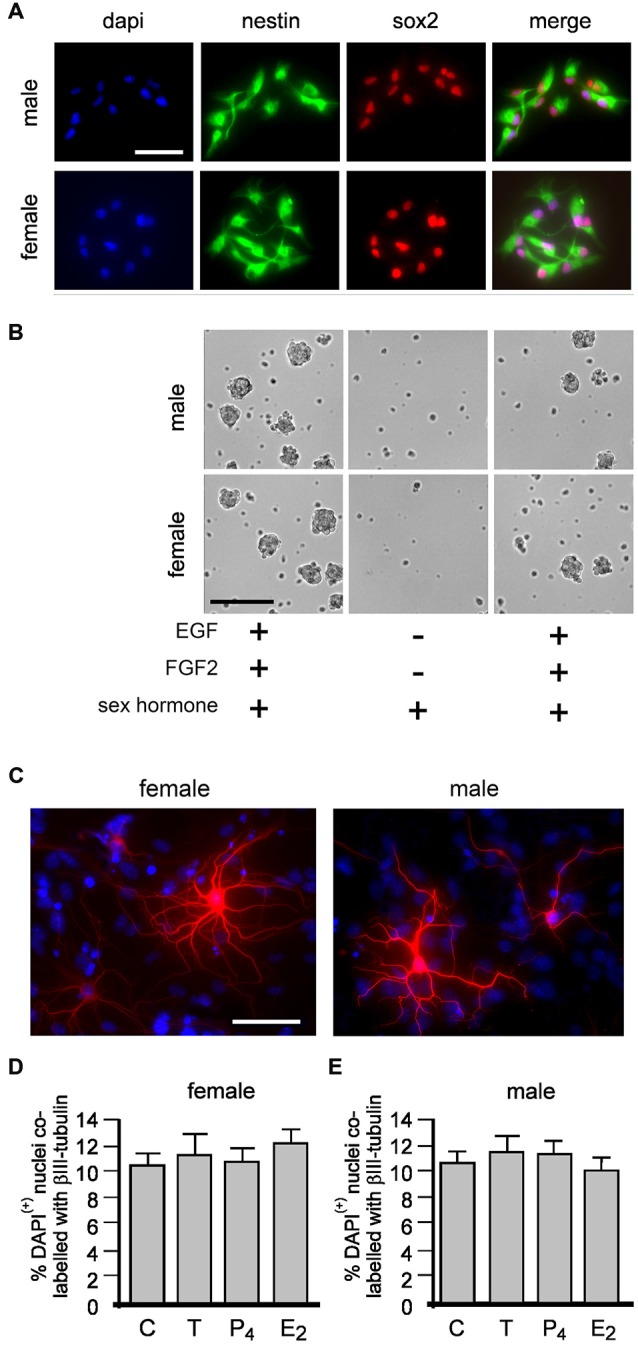
**Female and male neural stem/progenitor cells (NSPCs) were maintained as neurospheres for 10 passages without sex hormones or with either 10 nM of; testosterone (T), 17βestradiol (E_2_) or progesterone (P_4_).** At passages 2 and 10 neurospheres grown for 4 days from each group were allowed to adhere to a laminin substrate for 60 min, fixed and immunostained for neural stem cell markers nestin and sox2 (**A**, neurospheres maintained without any sex hormones at passage 2 shown). Female and male neurospheres grown for 1 passage with epidermal growth factor (EGF) and fibroblast growth factor-2 (FGF-2) with either 10 nM T, E_2_ or P_4_ (sex hormones) then passaged into media with the same sex hormone but without EGF or FGF showed a paucity in neurosphere formation, but re-addition of EGF and FGF stimulated neurosphere growth again (**B**, phase image of male and female neurospheres with T shown). Female and male neurospheres were maintained for five serial passages without sex hormones or with 10 nM T, E_2_ or P_4_ were then allowed to differentiate without any (*Continued*) sex hormones for 72 h when βIII-tubulin (red) immunocytochemistry was used to detect neuronal differentiation (**C**, neurons maintained without any sex hormones shown). No effect of maintenance in any condition was seen on the proportion of neuronal differentiation in female **(D)** or males **(E)**. Results show mean ±SEM. Scale bar = 50 μM **(A,C)** 150 μM **(B)**.

Our present study maintained neurospheres in the presence of potent mitogens EGF and FGF2 but without P_4_. Because P_4_ is a widely used standard component of neurosphere media, we first examined whether the absence of P_4_ affected expression of established markers of adult NSPCs, nestin and sox2 (Doetsch et al., 1999; Faigle and Song, 2013). Neurospheres grown for 4 days at passages 2 and 10 maintained ± 10 nM of T or P_4_ or E_2_ were allowed to adhere to a laminin substrate for 1 h before fixation. Subsequent immunocytochemistry showed all cells (DAPI+ nuclei) expressed both nestin and sox2 irrespective of sex or which sex hormone they were maintained in for up to 10 passages (Figure 2A). We next tested whether equal molar concentrations of T, E_2_ or P_4_ could independently maintain neurosphere growth without EGF and FGF2. Neurospheres grown over 7 days in standard proliferation media containing EGF and FGF were passaged into media with either 10 nM T, E_2_ or P_4_ without EGF or FGF where we observed an overt paucity in neurosphere formation over a subsequent 7 days (Figure 2B). However, neurosphere growth resumed following re-passaging into standard proliferation media containing EGF and FGF (Figure 2B).

Next we maintained male and female neurospheres over five serial passages ± 10 nM T, E_2_ or P_4_. On the 6th passage we maintained neurospheres from *all* treatments in media without sex hormones for 4 days. These neurospheres were then allowed to adhere to a laminin substrate and differentiated *without* sex hormones for 2 days then assessed for neuronal differentiation using βIII tubulin immunocytochemistry (Figure 2C). One-way ANOVA comparison of neuronal differentiation showed no change between neurospheres maintained without sex hormones or with either T, P_4_ or E_2_ in females (Figure 2D), *F*_(3,8)_ = 0.3803, *p* = 0.7700, and males, *F*_(3,8)_ = 0.2932, *p* = 0.8293 (Figure 2E).

### Sex Hormone Effects on NSPC Proliferation and Neuronal Differentiation

**Figure 3:**
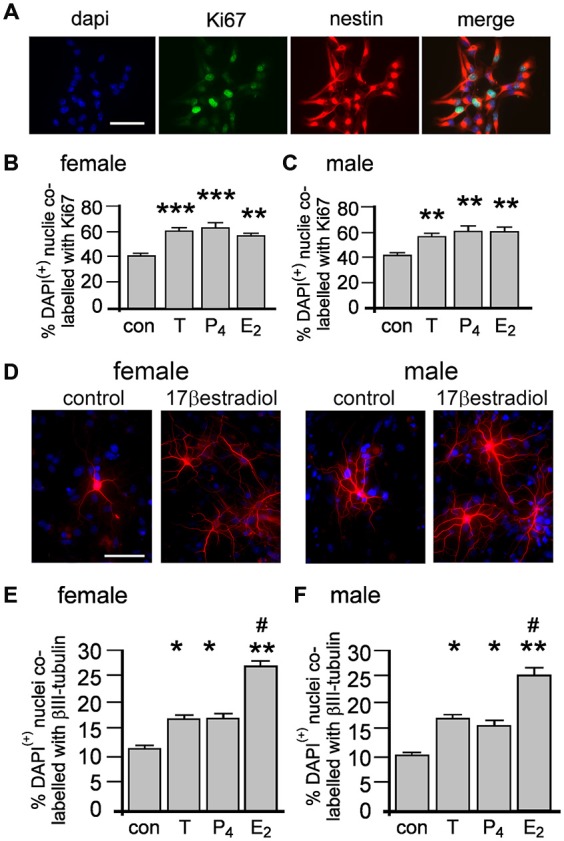
**Female and male NSPCs were grown as neurospheres without sex hormones for 4 days then allowed to adhere to a laminin substrate and expose to either 10 nM testosterone (T), 17β-estradiol (E_2_), progesterone (P_4_) for 20 min before Ki67/nestin double label fluorescent immunocytochemistry was performed (A, female control neurosphere shown).** The proportion of DAPI/nestin-labeled cells expressing Ki67 per neurosphere was increased by 20 min of either T, E_2_ or P_4_ compared to controls in both females **(B)** and males **(C)**. Female and male neurospheres maintained without sex hormones were differentiated on a laminin substrate for 72 h ± 10 nM T, E_2_ or P_4_ when βIII-tubulin (red) immunocytochemistry **(D)** showed that while T, P_4_ and E_2_ increased the proportion of neuronal differentiation compared to controls, E_2_ was more potent compared to T and P_4_ in females **(E)** and males **(F)**. Results show mean ± SEM, *p < 0.05, **p < 0.01, ***p < 0.005 compared to control, ^#^*p* < 0.05 compared to T and P_4_, one-way ANOVA with Tukey’s multiple comparisons test. Scale bar = 50 μM.

We adopted an immunocytochemical approach utilizing the intrinsic cell proliferation marker Ki67 to assess acute proliferative effects on nestin(+) NSPCs (Figure 3A). Neurospheres grown for 4 days in standard proliferation media were allowed to adhere to a laminin substrate for 60 min and then treated with 10 nM of either T, E_2_ or P_4_ for 20 min then fixed for immunocytochemistry. Neurospheres typically flattened and separated but remained within a defined margin. We randomly sampled adherent neurospheres addressing possible bias of size by restricting analyses to neurospheres containing 15–25 DAPI/nestin (+) cells. In female cultures one-way ANOVA showed a statistically significant main effect of treatment on the percentage of DAPI/nestin(+) cells expressing Ki67 per neurosphere, *F*_(3,8)_ = 20.15, *p* < 0.001 (Figure 3B). Tukey’s multiple comparison test showed that T, E_2_ and P_4_ independently increased proliferation compared to control, **p < 0.01, ***p < 0.005, (Figure 3B), but showed no significant differences between T, E_2_ or P_4_. The same analyses in male neurospheres demonstrated similar results with one-way ANOVA showing a statistically significant main effect of treatment on the percentage of DAPI/nestin(+) cells expressing Ki67 per neurosphere, *F*_(3,8)_ = 16.59, *p* < 0.001 (Figure 3C). Again Tukey’s multiple comparison test showed that T, E_2_ or P_4_ independently increased proliferation compared to control in males but showed no change between each hormone, **p < 0.01, (Figure 3C). Female and male neurospheres maintained without sex hormones in standard proliferation media were allowed to adhere to a laminin substrate and differentiate for 2 days in differentiation media ± 10 nM T, E_2_ or P_4_ whereupon βIII tubulin immunocytochemistry was used to assess neuronal differentiation (Figure 3D). One-way ANOVA showed a significant main effect of treatment in female neurospheres, *F*_(3,8)_ = 45.59, *p* < 0.0001 and Tukey’s multiple comparisons test showed that compared to control: T, *p < 0.05; P_4_, *p < 0.05 and E_2_, **p < 0.01 all increased neuronal differentiation, while E_2_ also showed a significant effect compared to T and P_4_, both #*p* < 0.001 (Figure 3E). An almost exact pattern was found when we performed one-way ANOVA on male neurosphere data; a significant main effect of treatment, *F*_(3,8)_ = 40.58, *p* < 0.0001 and Tukey’s multiple comparisons test showed that compared to control: T, *p < 0.05; P_4_, *p < 0.05 and E_2_, **p < 0.001 all increase neuronal differentiation, while E_2_ also showed a significant effect compared to T and P_4_, both #*p* < 0.001, (Figure 3F).

### Sex Hormone Effects on Neurosphere Growth

**Figure 4:**
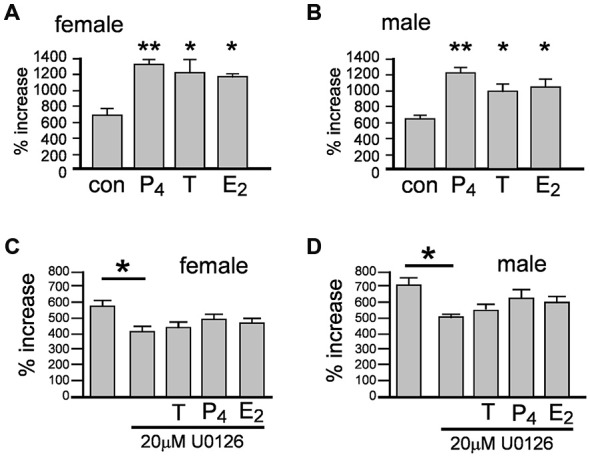
**Female and male neurospheres were grown for 7 days ± 10 nM of either testosterone (T), 17β-estradiol (E_2_), progesterone (P_4_).** Each sex hormone significantly increased neurosphere growth to the same degree compared to controls in females **(A)** and males **(B)** Over a 7 day period the Erk inhibitor U0126 significantly impaired neurosphere growth compared to controls and abolished the increase in response to each sex hormone in females **(C)** and males **(D)**. Results show mean ± SEM, **p < 0.05, **p < 0.01 compared to controls, one-way ANOVA with Tukey’s multiple comparisons test.

We next examined whether the acutely induced proliferation described in Figures 3A,B enhanced neurosphere growth by measuring the percentage increase in NSPCs after 7 days ± 10 nM T, E_2_ or P_4_ (Figures 4A,B). One-way ANOVA of female cultures showed a significant main effect of treatment on percentage increase in NPSCs, *F*_(3,8)_ = 8.46, *p* < 0.001, (Figure 4A). Tukey’s multiple comparison test showed that T, E_2_ and P_4_ independently increased neurosphere growth compared to control, *p < 0.05, **p < 0.01, without any significant difference between each of the sex hormones (Figure 4A). In male cultures one-way ANOVA showed the same pattern with a significant main effect of treatment on percentage increase in NPSCs, *F*_(3,8)_ = 10.95, *p* < 0.01 and Tukey’s multiple comparison test showing T, E_2_ and P_4_ independently increased neurosphere growth compared to control, *p < 0.05, **p < 0.01, without any significant difference between each sex hormone, (Figure 4B).

The acute proliferative effects of T, E_2_ or P_4_ on NSPCs also prompted us to consider the involvement of rapid, non-classical steroid signaling. Several well characterized rapid signal-transduction pathways have been implicated in sex hormone actions, including extracellular-regulated kinase pathways such as MEK1-Erk, which also play regulatory functions in NSPCs (Simoncini and Genazzani, 2003; Faigle and Song, 2013). The protein kinase inhibitor U0126 can inhibit MEK1-stimulated Erk phosphorylation at 10 μM and has been used up to 30 μM to examine neurosphere properties, including inhibition of FGF2-stimulated Erk phosphorylation (Davies et al., 2000; Jin et al., 2005; Bain et al., 2007; Dottori et al., 2008). We tested the ability of T, E_2_ or P_4_ at equal molar concentrations to induce neurosphere growth over 7 days with concomitant MEK1-Erk signaling inhibition using 20 μM U0126. In female cultures one-way ANOVA showed a significant main effect, *F*_(4,10)_ = 3.72, *p* < 0.05, with Tukey’s multiple comparisons test showing that neurosphere growth was significantly attenuated by 20 μM U0126 compared to controls, *p < 0.05, (Figure 4C). Once again the pattern of statistical analyses results were the same in male cultures; one-way ANOVA showed a significant main effect, *F*_(4,10)_ = 4.73, *p* < 0.05, with Tukey’s multiple comparisons test showing significantly attenuated of neurosphere growth by 20 μM U0126 compared to controls, *p < 0.05, (Figure 4D). Comparisons of U0126 + sex hormones to either controls or U0126 alone showed no statistically significant changes in either sexes indicating U0126 impaired the capacity of each sex hormone to induce neurosphere growth, (Figures 4C,D).

### Differentiating Between Androgen and Estrogen Effects on Neurosphere Growth

**Figure 5:**
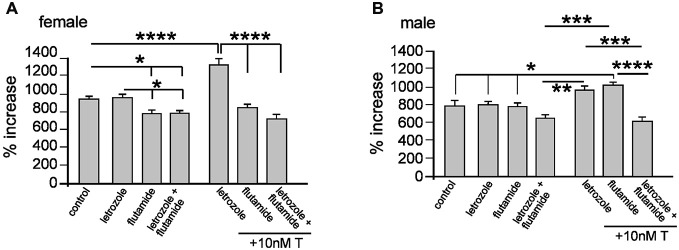
**Female and male neurospheres were grown for 7 days ± 10 μM of the aromatase inhibitor letrozole, 10 μM of the androgen receptor antagonist flutamide ± 10 nM testosterone (T) as indicated.** Letrozole alone did not affect female of male neurosphere growth. Flutamide alone or with letrozole impaired female neurosphere growth **(A)**, while in males only the combined addition of letrozole and flutamide reduced growth **(B)**. Co-addition of T induced female neurosphere growth in the presence of letrozole but not flutamide or letrozole/flutamide combined **(A)** and in males only combined letrozole/flutamide significantly reduced growth compared to either inhibitor alone. Results show mean ± SEM, *p < 0.05, **p < 0.01, ***p < 0.005, ****p < 0.001, one-way ANOVA with Tukey’s multiple comparisons test.

Aromatase is an enzyme catalyzing the conversion of T into E_2_ and one report has shown aromatase expression in adult rat-derived neurospheres (Boon et al., 2010; Waldron et al., 2010b). The implication for cells expressing aromatase is that exogenous T, through *metabolism* into E_2_, could act through ER, in addition to its cognate AR. We used a pharmacological approach to examine whether exogenous T promotes neurosphere growth through estrogenic or androgenic pathways, employing the specific aromatase inhibitor *letrozol* and AR antagonist *flutamide*. These compounds have high specificity, and used in human cancer cell lines show for example, decreased E_2_ synthesis and anti-proliferative effects of letrozole at concentrations ranging from 10 nM to 100 μM (Bhatnagar et al., 1990; Brogden and Chrisp, 1991; Mitropoulou et al., 2003; Han et al., 2008). We examined female neurosphere growth in the presence of letrozole and flutamide alone or combined and ± 10 nM T, with one-way ANOVA showing a significant main effect of treatment, *F*_(6,14)_ = 39.83, *p* < 0.001 (Figure 5A). Tukey’s multiple comparison test showed that compared to control neurosphere growth, addition of letrozole alone had no effect, while flutamide with or without letrozole significantly inhibited growth, *p < 0.05, (Figure 5A). When 10 nM T was added with letrozole, neurosphere growth was increased significantly compared to all other groups, ****p < 0.001, while 10 nM T applied with flutamide alone or flutamide and letrozole did not stimulate growth (Figure 5A). In male neurospheres, we observed a different pattern of results. One-way ANOVA demonstrated a significant main effect of treatment, *F*_(6,14)_ = 13.08, *p* < 0.001 (Figure 5B). However, contrasting the effects on female neurosphere growth Tukey’s multiple comparison test showed that compared to controls 10 nM T increased growth in the presence of flutamide, *p < 0.05, but not letrozole (Figure 5B). Additionally, neurosphere growth when 10 nM T was applied in the presence of both letrozole and flutamide was significantly reduced compared to either inhibitor alone + 10nM T, ***p < 0.005 (letrozole alone), ****p < 0.001 (flutamide alone) (Figure 5B).

### Oxidative Stress, Neurosphere Growth and Aromatase Expression

**Figure 6:**
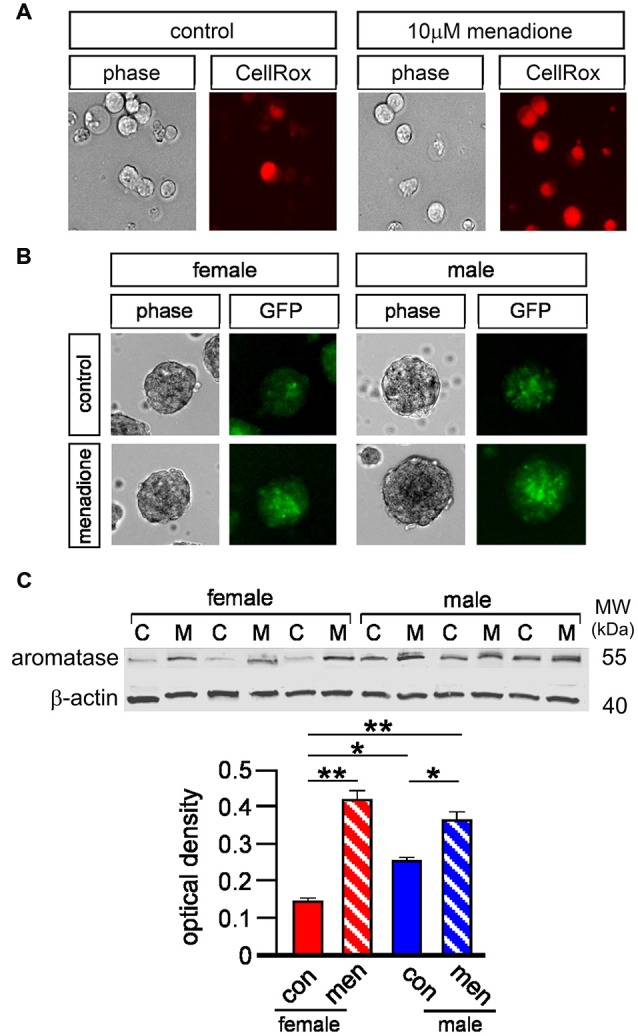
**Five micromolar CellROX orange™ was used with live-cell imaging to fluorescently detect basal and menadione-induced levels of ROS in female and male neurospheres (A, panels show example of female neurospheres incubated ±10 μM menadione for 48 h).** Neurospheres derived from GFP-aromatase transgenic mice were grown for 5 days under control conditions then briefly imaged by both light microscopy and epi-fluorescence (control) then after another day challenged with 10 μM menadione for 48 h and imaged again qualitatively showing higher basal aromatase expression in males compared to females and also menadione-induced aromatase expression in both sexes **(B)**. Total cell protein was isolated from male and female neurospheres grown ± 10 μM menadione for 5 h [C = control (con), M = menadione (men)] and resolved by SDS-PAGE and probed for aromatase and β actin **(C)**. Quantitative densitometry analyses showed significantly higher levels of aromatase in male compared to female neurospheres under controls conditions and a significant increase in aromatase expression in both sexes after menadione treatment **(C)**. Results in C show mean ± SEM, *p < 0.01, **p < 0.001, two-way ANOVA with Tukey’s multiple comparisons test.

Local aromatase-mediated E_2_ synthesis is thought to be induced by brain insults through mechanisms such as oxidative stress (Garcia-Segura et al., 2003; Cornelius et al., 2013). This, together with Figure 5 data showing sex-specific effects of letrozole, prompted us to examine aromatase expression in adult NSPCs. Firstly, we used a commercial fluorescent marker of reactive oxygen species (ROS) to demonstrate that 10 μM menadione—a compound widely used to model oxidative stress *in vitro* (Morrison et al., 2011)—induced ROS in cultured neurospheres (Le Belle et al., 2011; Figure 6A). Next we derived neurospheres from adult male and female transgenic mice that express a green fluorescent protein (GFP)—*Cyp19* construct (Stanić et al., 2014). These mice express GFP under the same physiological promoter control as the *Cyp19* gene that encodes aromatase, such that GFP is a reliable reporter of aromatase expression. Male and female GFP-aromatase neurospheres grown for 5 days were imaged with live-cell phase and fluorescent microscopy then after a further day challenged for 24 h with 10 μM menadione to induce formation of ROS (Loor et al., 2010) and imaged again (Figure 6B). Randomly examining 40–50 imaged neurospheres per condition appeared to qualitatively demonstrate higher basal levels of GFP fluorescence in males compared to females indicating higher aromatase expression. Furthermore, menadione appeared to induce GFP fluorescence in neurospheres of both sexes (Figure 6B). To substantiate this qualitative observation, we next isolated total cell protein from female and male neurospheres for quantitative Western blot. Whole cell lysates were derived from control neurospheres and those challenged with 10 μM menadione for 3 h and proteins resolved by SDS-PAGE and probed with an aromatase antibody that detected a band at approximately 55 kilo-Daltons the expected molecular weight (Figure 6C). Subsequent densitometry analyses using two-way ANOVA showed a significant interaction between sex and treatment, *F*_(1,8)_ = 24.28, *p* < 0.001 and a significant main effect of treatment, *F*_(1,8)_ = 133.4, *p* < 0.0001 (Figure 6C). Multiple comparisons Tukey’s tests showed under control conditions male neurospheres had higher aromatase levels compared to females, *p < 0.01 and that menadione-induced oxidative stress increased aromatase expression in females, **p < 0.001 control vs. menadione and males, *p < 0.01 control vs. menadione (Figure 6C).

**Figure 7:**
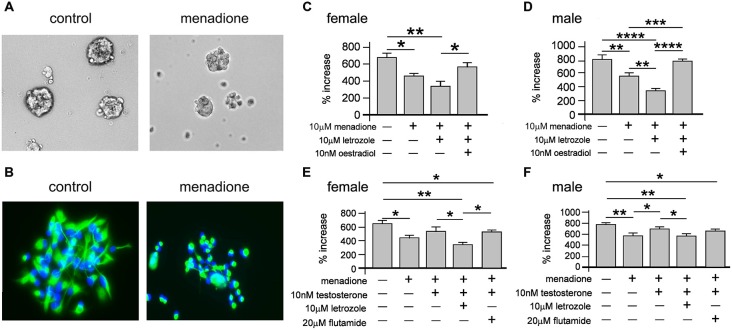
**Neurospheres displayed reduced size when grown in the presence of 10 μM menadione (A, example show male neurospheres ± menadione for 5 days).** Neurospheres grown for 5 days ± 10 μM menadione were allowed to adhere to a laminin substrate for 1 h then fluorescently stained with DAPI (blue) and an antibody to nestin (green) showing that oxidative stress disrupted nestin(+) morphology (**B**, example shows male neurospheres). Neurosphere growth over 7 days was significantly impaired by 10 μM menadione alone compared to controls but not with concomitant exogenous addition of 10 nM E_2_ in females **(C)** and males **(D)**. However, inhibiting intrinsic E_2_ synthesis with letrozole significantly exacerbated the menadione-induced impaired growth in males **(D)** but not females **(C)**. An independent set of experiments showed addition of 10 nM exogenous testosterone could abolish menadione-impaired neurosphere growth compared to controls, which was prevented with the co-addition of letrozole but not flutamide in females **(E)** and males **(F)**. Results show mean ± SEM, *p < 0.05, **p < 0.01, ***p < 0.005, ***p < 0.001, one-way ANOVA with Tukey’s multiple comparisons test.

We next tested how oxidative stress effected neurosphere growth over 7 days. Neurospheres of both sexes grown with 10 μM menadione showed comparative smaller sizes by 5 days (Figure 7A). We also immunostained adherent female and male neurospheres after 5 days ± 10 μM menadione to show an overt impairment in nestin(+) morphology (Figure 7B). We then assessed female and male neurosphere growth over 7 days ± 10 μM menadione alone or in combination with 10 μM letrozole or 10 nM E_2_ (Figures 7C,D). One-way ANOVA showed a significant main effect of treatment in female neurospheres, *F*_(3,8)_ = 10.33, *p* < 0.005, with Tukey’s multiple comparison test showing that menadione alone, *p < 0.05 and menadione + letrozole, **p < 0.01 decreased neurosphere growth compared to control, while a significant difference in neurosphere growth between menadione + letrozole and menadione + E_2_ was also evident, *p < 0.05 (Figure 7C). One-way ANOVA of male neurosphere data also showed a significant main effect of treatment, *F*_(3,8)_ = 50.46, *p* < 0.001 (Figure 7D). Tukey’s multiple comparison test in males showed a similar pattern to females, however in males the combination of menadione + letrozole significantly decreased neurosphere growth compared to menadione alone, **p < 0.01 (Figure 7D). Of important note E_2_ was able to rescue the menadione-inhibition of growth in both female and male neurospheres (Figures 7C,D).

We next assessed whether T could also rescue menadione-induced inhibition of female and male neurosphere growth (Figures 7E,F). One-way ANOVA of female neurosphere growth showed a significant main effect of treatment, *F*_(4,10)_ = 8.28, *p* < 0.005 (Figure 7E). Tukey’s multiple comparisons test replicated our previous data set showing menadione-induced inhibition of growth compared to controls, *p < 0.05, while 10 nM T added with menadione abolished this deficit (Figure 7E). However, when T was added to menadione-treated cultures with letrozole *or* flutamide then neurosphere growth remained diminished compared to controls, **p < 0.01 and *p < 0.05, respectively (Figure 7E). We observed a similar main effect of treatment in males with one-way ANOVA, *F*_(4,10)_ = 8.28, *p* < 0.005, and similar Tukey’s multiple comparison test results where 10 nM T alone rescued growth inhibition in menadione-treated cultures compared to controls, but not in the presence of either letrozole where growth was significantly reduced compared to controls, **p < 0.01, or flutamide, *p < 0.05 compared to control (Figure 7F).

## Discussion

Adult NSPCs are the principal source of ongoing adult neurogenesis, providing a mechanism of self-regeneration. Adult neurogenesis shows a capacity to change in response to altered levels of sex hormones, a possible basis for sex-dependent adult neurogenesis. Our present study has utilized neurospheres to compare equal molar effects of principal sex hormones on male and female NSPCs.

We demonstrate that T, E_2_ and P_4_ can each promote NSPC proliferation and neurosphere growth. Each sex hormone was able to enhance neurosphere growth to the same degree at equal molar concentrations. Of particular note is that all three sex hormones had the same effect irrespective of genetic sex. From an *in vivo* perspective of circulating concentrations, T is comparatively abundant compared to either E_2_ or P_4_ in males, whilst the opposite is true for premenopausal females. At the cellular level, *in vitro* at least, it appears that the genetic sex of NSPCs does not program proliferative responses to what are generally considered sex-specific hormones. This is perhaps not true for the mechanism by which T exerts its effects as discussed later. The capacity for T, E_2_ and P_4_ to promote NSPC proliferation was dependent on the presence of the potent mitogens EGF and FGF2. Removal of these factors resulted in a clearly evident paucity in neurosphere formation. We therefore suggest that the observed proliferative enhancement exerted by each sex hormone could be the result of potentiated EGF and FGF2 signal transduction.

**Figure 8:**
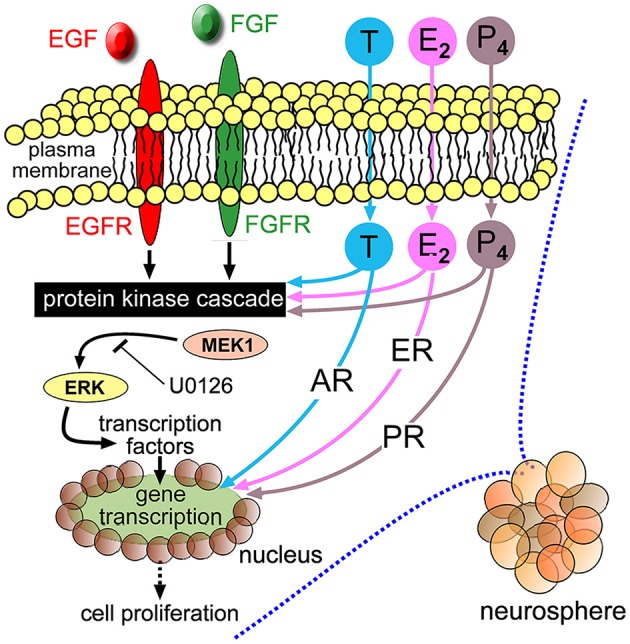
**Schematic diagram depicting EGF, fibroblast growth factor 2 (FGF2) and their respective transmembrane receptors (EGFR and FGFR) stimulating intracellular protein kinases, including MEK1-Erk, leading to cellular proliferation.** Extracellular T, E_2_ and P_4_ freely cross plasma membranes to bind intracellular cognate AR, ER or PR, respectively. The neurosphere growth-promoting effects of T, E_2_ and P_4_ could be mediated through potentiating protein kinase signaling stimulated by EGF and FGF2, where co-application of the MEK1 inhibitor U0126 impairs their respective potentiation effects. There may also be a contribution through classical steroid hormone signaling mediated by nuclear translocation of cognate receptors that interact with hormone response elements with resulting gene transcription.

Receptors for T, E_2_ and P_4_ belong to a related family of intracellular steroid nuclear receptors. “Classic” steroid hormone signaling describes the translocation of these receptors in complexes to the nucleus to modifying gene expression (Guerriero, 2009). This form of signaling is generally considered on a longer time-scale compared to signal-transduction utilizing rapid protein phosphorylation events. E_2_ in particular has been observed to produce rapid effects in neural tissue such as synapse modifications (Srivastava et al., 2011) and contributed to speculation of the existence of transmembrane receptors capable of transducing rapid signaling through protein phosphorylation. We suggest this mechanism could partly underlie the enhanced neurosphere growth we report here. U0126 is an inhibitor of MEK1-mediated Erk phosphorylation and cell-based assays show concentrations of 10 μM can suppress Erk phosphorylation and also abolish Erk phosphorylation stimulated by either EGF or FGF2 (Davies et al., 2000; Jin et al., 2005; Bain et al., 2007; Dottori et al., 2008). Using 20 μM U0126, we demonstrated a loss of the growth-promoting effects of each sex hormone. Coupled with our demonstration that NSPC-proliferation was stimulated within 20 min of sex hormone application, we suggest that T, E_2_ and P_4_ could potentiate protein kinase signaling induced by EGF and FGF2. This notion is strengthened when we consider independent studies have shown that T can stimulate Erk phosphorylation (Estrada et al., 2003) and that Mitogen-activated protein kinase (MAPK) signaling is instrumental in maintaining NSPC proliferation (Faigle and Song, 2013). Quantitative Western blot analyses could further substantiate the involvement of MEK1-Erk signaling in response to sex hormone stimulation, since upstream kinases other than MEK1 could still target Erk for phosphorylation. Furthermore, our current data cannot conclusively exclude a contribution at the level of AR or ER-mediated gene transcription. Transcriptional analyses of adult NSPCs in response to sex hormones as a function of genetic sex are ongoing areas of investigation in our laboratory. We have summarized a possible mechanism in Figure 8.

At a cellular level the liberation of mitochondrial cholesterol is the initial step in *de novo* sex hormone synthesis followed by a series of reactions catalyzed by specific steroidogenic enzymes (Miller and Auchus, 2011). An outstanding question emanating from our present study concerns determining whether adult NSPCs synthesize sex hormones *de novo*. However, demonstrating that AR inhibition in female neurospheres reduced their growth could suggest innate androgen synthesis participates in basal NSPC proliferation *in vitro* at least. In contrast, a reduction in basal male neurosphere growth was observed only when the AR and aromatase were concomitantly inhibited. A feature of cells expressing subsets of steroidogenic enzymes is their capacity to metabolize exogenous sex hormones. In principal then, the function of aromatase in NSPCs could be either innate E_2_ synthesis or exogenous T metabolism into E_2_. We observed that exogenous T stimulated female neurosphere growth when aromatase was inhibited but was dependent on the AR. This was not the case in male NSPCs where T promoted neurosphere growth in the presence of AR antagonism, but not when aromatase activity was inhibited. This presents an important sex-specific aspect to our current study; aromatase metabolism of T into E_2_ plays a role in male but not female NSPC proliferation. This idea is supported by previous studies reporting higher levels of aromatase in adult male neurospheres (Waldron et al., 2010b), which accords with our current data derived from Western blot protein analyses and also our new GFP-aromatase reporter model. Moreover, male bias for local aromatase expression reconciles with a “*male need*” for local E_2_ synthesis compared to females given the disparity in circulating E_2_ levels.

The physiological functions of brain aromatase remain unknown, however a capacity for local E_2_ synthesis to enhance synaptic plasticity and support injured neurons implies a possible neuroprotective role (Garcia-Segura et al., 2003). Neuroprotective properties are not fully sufficient to redress the inevitable neuronal cell death resultant from severe brain injury. Inducible expression in NSPCs could conceivably afford aromatase a neuro-restorative role as well. Such a notion is supported by experimental stroke models showing local aromatase-mediated E_2_ synthesis is required for injury (stroke)-induced neurogenesis (Li et al., 2011). We employed a model of oxidative stress primarily because this pathology is common in neurodegenerative diseases, traumatic brain injuries and stroke (Cornelius et al., 2013). Aromatase expression increased in neurospheres with raised levels of ROS demonstrating in principal that trauma or injury could induce innate E_2_ synthesis in NSPCs. Although oxidative stress impaired neurosphere growth in both sexes, concomitant aromatase inhibition exacerbated the impairment, primarily in male neurospheres. While induced cellular E_2_ synthesis alone appeared insufficient to fully block ROS-mediated growth impairment, aromatase nonetheless attenuated the impairment. We also examined relative androgenic and estrogenic actions of exogenous T on its ability to rescue oxidative stress-impaired neurosphere growth. Of important note, when T was given alone it could significantly, though mildly, reverse the ROS-induced reduction in neurosphere growth in both sexes. However, if aromatase-mediated *metabolism* of T into E_2_ was inhibited then this rescue effect of exogenous T was lost. The collective oxidative stress data allude to the notion that androgenic actions of T could be detrimental under pathological conditions and may explain why induced aromatase-mediated E_2_ synthesis was not fully protective. This idea has been postulated previously, though the effects of androgens in the pathological brain remain equivocal (Ransome, 2012a; Quillinan et al., 2014).

A key ability of inducible aromatase expression is to control the relative androgenic and estrogenic signaling at the cellular level. Recent clinical data from severe traumatic brain injury patients assessed the ratio of E_2_ to T levels in the cerebrospinal fluid (CSF) in relation to patient outcomes. This study provided two key points, firstly it measured sex hormones in the CNS environment (i.e., CSF) rather than circulating levels to show high E_2_ to T ratio was associated with better outcomes compared to a low ratio. Secondly, it showed a co-association of this effect with polymorphisms in the aromatase gene. Moreover, we suggest that aromatase could play *both* a role in neuroprotection *and* neuro-restoration by supporting NSPCs under pathological conditions. Our current study focused primarily on examining neurosphere growth through NSPC proliferation, which plays an essential role in expanding neural progenitor cells in the normal and pathological environment. Another question that waits empirical testing is whether aromatase supports neural differentiation and survival under pathological conditions. We are currently exploring this hypothesis and whether pharmacological promotion of estrogenic signaling at the expense of androgenic signaling is an efficacious strategy to replace the inevitable death of neurons in acquired brain injuries.

## Concluding Remarks

We used isolated adult NSPCs to demonstrate a direct response to principal sex hormones irrespective of genetic sex. However, while T acts substantively through the AR in female NSPCs, male response to T could be elicited by either AR or ER as a consequence of higher aromatase expression. Given that T is synonymous with male sexual differentiation, a male bias in the aromatase-mediated E_2_ synthesis is counter intuitive. However, this notion is reconciled if we propose that E_2_ plays fundamental non-reproductive roles in both sexes. Hence the male profile of comparatively low circulating E_2_ but high T would be consistent with higher levels of localized aromatase expression. We additionally provide evidence that aromatase-mediated E_2_ synthesis could support the innate NSPCs response in brain injuries. Our future studies will aim to trace aromatase expression in differentiating progeny of adult NSPCs to examine ways of supporting neuronal production under pathological conditions.

## Conflict of Interest Statement

The authors declare that the research was conducted in the absence of any commercial or financial relationships that could be construed as a potential conflict of interest.
